# Quality of Life, Toxicity and Unmet Needs in Nasopharyngeal Cancer Survivors

**DOI:** 10.3389/fonc.2020.00930

**Published:** 2020-06-12

**Authors:** Lachlan McDowell, June Corry, Jolie Ringash, Danny Rischin

**Affiliations:** ^1^Department of Radiation Oncology, Peter MacCallum Cancer Centre, Melbourne, VIC, Australia; ^2^Sir Peter MacCallum Department of Oncology, The University of Melbourne, Melbourne, VIC, Australia; ^3^GenesisCare Radiation Oncology, Division Radiation Oncology, St. Vincent's Hospital, Melbourne, VIC, Australia; ^4^Department of Medicine St Vincent's, The University of Melbourne, Melbourne, VIC, Australia; ^5^Department of Radiation Oncology, Princess Margaret Cancer Centre/University of Toronto, Toronto, ON, Canada; ^6^Department of Medical Oncology, Peter MacCallum Cancer Centre, Melbourne, VIC, Australia

**Keywords:** nasopharyngeal carcinoma, radiotherapy, chemotherapy, quality of life, survivorship, toxicity, unmet needs

## Abstract

Concerted research efforts over the last three decades have resulted in improved survival and outcomes for patients diagnosed with nasopharyngeal carcinoma (NPC). The evolution of radiotherapy techniques has facilitated improved dose delivery to target volumes while reducing dose to the surrounding normal tissue, improving both disease control and quality of life (QoL). In parallel, clinical trials focusing on determining the optimal systemic therapy to use in conjunction with radiotherapy have been largely successful, resulting in improved locoregional, and distant control. As a consequence, neoadjuvant chemotherapy (NACT) prior to definitive chemoradiotherapy has recently emerged as the preferred standard for patients with locally advanced NPC. Two of the major challenges in interpreting toxicity and QoL data from the published literature have been the reliance on: (1) clinician rather than patient reported outcomes; and (2) reporting statistical rather than clinical meaningful differences in measures. Despite the lower rates of toxicity that have been achieved with highly conformal radiotherapy techniques, survivors remain at moderate risk of persistent and long-lasting treatment effects, and the development of late radiation toxicities such as hearing loss, cranial neuropathies and cognitive impairment many years after successful treatment can herald a significant decline in QoL. Future approaches to reduce long-term toxicity will rely on: (1) identifying individual patients most likely to benefit from NACT; (2) development of response-adapted radiation strategies following NACT; and (3) anticipated further dose reductions to organs at risk with proton and particle therapy. With increasing numbers of survivors, many in the prime of their adult life, research to identify, and strategies to address the unmet needs of NPC survivors are required. This contemporary review will summarize our current knowledge of long-term toxicity, QoL and unmet needs of this survivorship group.

## Introduction

The last three decades have seen considerable progress in the management of patients diagnosed with nasopharyngeal cancer (NPC). While highly conformal radiotherapy (RT) remains the backbone of NPC treatment, the main research focus has been on optimal integration of systemic therapy for both radiosensitization to maximize locoregional control and eradication of micrometastatic disease to address the predominant mode of treatment failure (1, 2). The landmark intergroup 0099 study (3) established concomitant and adjuvant cisplatin-based chemotherapy as the standard of care, although much controversy followed, spawning a generation of clinical trials and meta-analyses targeted at identifying those patients most likely to derive benefit from additional systemic treatment (4–8). Flash forward to 2020 and neoadjuvant chemotherapy (NACT) in combination with platinum-based concurrent chemoradiation has become the preferred approach (9–12). In parallel, major technological advances in radiotherapy delivery have allowed for dose escalation to target volumes and reduced normal tissue dosing, resulting in improved disease control (13, 14), toxicity, and QoL (13–19).

While these successes should be celebrated, NPC survivors still harbor a substantial burden of long-term toxicity following successful treatment of their cancer. In endemic populations, the incidence of NPC shows a sharp increase from the third decade, peaking in the sixth decade; in low-risk populations, the incidence increases with age (20). As a consequence, the majority of patients afflicted with NPC are healthy middle-aged adults in the prime of their lives. While the most common and readily apparent radiation-induced toxicities of hearing loss, xerostomia, dysphagia, and hypothyroidism are well-quantified in the literature, survivors face numerous challenges. These include, amongst others, cognitive changes, fatigue, and emotional distress. Although targeted research is lacking, it is likely that NPC survivors will suffer from similar unmet needs to the general head and neck cancer (HNC) population, including workplace rehabilitation, sexual dysfunction and fear of cancer recurrence (21). The focus of this article will be to review our understanding of long-term toxicity, QoL and unmet needs and offer future avenues for targeted research in NPC populations.

## Toxicity and Quality of Life Outcomes

Much of our understanding of toxicities from prospective clinical trials has been reported through the eye of the clinician (Table 1). In contemporary oncology practice, the emphasis has shifted to patient-reported outcome measures (PROMs) as it is reasonably well-established that clinicians may unintentionally under report symptoms (46, 47) and their severity (48, 49) compared to patients. Only a few of the reported prospective series to date have included PROMs, including QoL assessments (Table 1). The most common PROM collected has been QoL, with the majority of studies using the validated European Organization for Research and Treatment of Cancer quality of life core questionnaire (EORTC QLQ-C30) and the accompanying head and neck module (EORTC QLQ- H&N35) (Table 1). The QLQ-C30 contains 30 items which map to a global health status/QoL score (composed of two items), five functional scales, three multi-item symptom scales, and six single symptom items (50); while the 35 items from the QLQ-H&N35 maps to seven symptom scales and 11 single items (51). Higher scores in the global QoL score and functional scales reflect better QoL or function, while on the symptom scales and items higher scores indicate an increased severity of symptomology. An additional challenge in interpreting QoL data in the current NPC literature has been the reliance on reporting statistically-determined differences rather than clinically meaningful differences, a limitation which has been identified across the oncology trials landscape (52). A commonly used “minimal clinically important difference” in the EORTC modules has been a difference of 10 (53), while Cocks and colleagues have ascribed numerical differences in the components of the EORTC QLQ-C30 as clinically trivial, small, medium or large (54). In the discussion that follows, the clinical difference proposed by Cocks et al. will be used in discussion on the QLQ-C30, and given there are no corresponding thresholds for the QLQ-H&N35, an estimate of 10 will be used to suggest a clinically meaningful differences in the components of that module.

**Table 1:** Selected NPC studies reporting long-term quality of life and toxicity and the instruments used.

**Study**	**No**.	**Treatment**	**RT**	**QoL assessment**	**Toxicity measurement**	
					**Clinician-reported**	**Patient-reported**
**PROSPECTIVE CONCURRENT CRT** **±** **NEO/ADJUVANT CHEMOTHERAPY**						
Sun et al. (10) Li et al. (22)	241	HDC-CRT ± TPF induction	IMRT 100%	Not yet reported	Acute - CTCAE v3.0 Late - RTOG	No
Tan et al. (23)	172	LDC-CRT ± GCP induction	IMRT 98% 2D 4%	EORTC QLQ-C30 and H&N35	Acute - CTCAE RTOG Late - RTOG	EORTC QLQ-H&N35
Zhang (9)	480	HDC-CRT ± GC induction	IMRT 100%	No	Acute - CTCAE v4.0 Late - RTOG	No
Cao (11)	476	MDC-CRT ± PF induction	IMRT 43% 2D 57%	No	Acute - CTCAE v4.0 Late - RTOG	No
Hui (12)	65	LDC-CRT ± DP induction	3D	EORTC QLQ-C30 and H&N35	Acute - CTCAE v2.0 Late RTOG	EORTC QLQ-H&N35
Hong (24)	479	LDC-CRT ± MEPFL induction	IMRT 61% 3D 39%	No	Acute - CTCAE v3.0 Late RTOG	No
Fountzilas (25)	141	LDC-CRT ± CET induction	3D 100%	No	Acute - CTCAE v3.0 RTOG - Late RTOG	No
Frikha (26)	83	LDC-CRT ± TPF	IMRT 37%	No	CTCAE v3.0	No
Chen (6, 27)	508	LDC-CRT ± PF	IMRT 42% 3D 5% 2D 53%	No	Chemotherapy toxicity - CTCAE v3.0 RT toxicity - RTOG	No
**PROSPECTIVE RT ALONE** **±** **CONCURRENT WITH ADJUVANT OR ADJUVANT STUDIES**						
Al-Sarraf et al. (3)	193	RT alone vs. HDC-CRT + PF	2D	No	SWOG	No
Wee et al. (4)	221	RT alone vs. HDC-CRT + PF	2D	No	Acute–RTOG	No
Lee et al. (28, 29)	348	RT alone vs. HDC-CRT + PF	2D 41% Mix 8% Conformal 61%	No	RTOG Chemo toxicities - WHO	No
Rossi et al. (30)	229	RT alone ± VAC	2D	No	Method/System NR	No
Chi et al. (31)	157	RT alone ± PF	2D	No	Method/System NR	No
**OTHER PROSPECTIVE CHEMOTHERAPY STUDIES**						
Chen, Li (5, 22)	230	RT alone vs LDC-CRT (stage II only)	2D	No	Acute–CTCAE Late–RTOG	No
Lee (2)	109	Weekly vs. triweekly CRT (+ Adjuvant)	IMRT 74% 2D-CRT 16%	EORTC QLQ-C30 and H&N35	Acute–RTOG	EORTC QLQ-H&N35
**PROSPECTIVE INTENSITY-MODULATED vs. 2D/3D RADIOTHERAPY STUDIES**						
Kam et al. (32)	60	IMRT vs. 2D	IMRT 50% 2D 50%	No	RTOG	No
Pow et al. (33)	46	IMRT vs. 2D	IMRT 52% 2D 48%	EORTC QLQ-C30 and H&N35	No	EORTC QLQ-H&N35
Peng et al. (14)	616	IMRT vs. 2D	IMRT 50% 2D 50%	No	CTCAE v3.0	No
Fang et al. (34)	203	IMRT vs. 3D	IMRT 46% 3DCRT 54%	EORTC QLQ-C30 and H&N35	No	EORTC QLQ-H&N35
**OTHER ENDEMIC STUDIES**						
Fang et al. (17)	237	28% CRT	Conventional 64% (2D 26%, 2D + 3D boost 38%) Conformal 36% (3D 14%, IMRT 22%	EORTC QLQ-C30 and H&N35	No	EORTC QLQ-H&N35
Fang et al. (35)	68	CRT 100%	VMAT 100%	EORTC QLQ-C30 and H&N35	CTCAE v4.03	EORTC QLQ-H&N35
Hong et al. (36)	216	CRT 98%	IMRT 75%	EORTC QLQ-C30	Reported, but method/system not clear	Reported, but method/system not clear
Fang et al. (18)	356	CRT 35%	IMRT 24% 3D 16% 2D+3D boost 30% 2D 30%	EORTC QLQ-C30 and H&N35	No	EORTC QLQ-H&N35
Pan et al. (37)	106	CRT 48%	IMRT 56% 2D 44%	EORTC QLQ-C30 and H&N35	No	EORTC QLQ-H&N35
Tsai et al. (19)	242	CRT 66%	IMRT 41% Non-IMRT 59%	EORTC QLQ-C30	CTCAE v4.0	No
Wu et al. (38)	192	CRT 23%	2D 34% Co-60 66%	Chinese SF-36	No	No
Lee et al. (13)	1593	-	IMRT 28% 3D 45% 2D 27%	No	RTOG CTCAE v3.0	No
**NON-ENDEMIC STUDIES**						
Tonoli et al. (39)	136	CRT 91%	IMRT 95% 3D 5%	No	CTCAE v3.0	No
McDowell et al. (40)	107	CRT 93%	IMRT 100%	FACT-HN	CTCAE v4.03	MDASI-HN
Takiar et al. (41)	66	CRT 99%	IMRT 100%	No	CTCAE v4.0	No
Lastrucci et al. (42)	25	CRT 64%	IMRT 16% 3D 21% 2D 63%	FACT-NP XeQoLS	CTCAE v4.03	No
Talmi et al. (43)	28	CRT 64%	2D	(UW)-QoL	No	(UW)-QoL includes patient reported toxicity
Ghiggia et al. (44)	21	CRT 100%	NS	EORTC QLQ-C30 and H&N35	RTOG	EORTC QLQ- H&N35
Yee et al. (45)	82	CRT 77%	3D 43% 2D 57%	No	RTOG	No

*RT, radiotherapy; QoL, Quality of Life; HDC, high dose/three weekly cisplatin; CRT, concurrent chemoradiotherapy; TPF, paclitaxel, cisplatin, 5-fluorouracil; IMRT, intensity-modulated radiotherapy; CTCAE, common terminology criteria of adverse events; RTOG, Radiation Therapy Oncology Group; LDC, low dose/weekly cisplatin; GCP, gemcitabine, carboplatin, paclitaxel; 2D, two dimensional radiotherapy; EORTC QLQ-C30, European organization for research and treatment of cancer core quality of life questionnaire; EORTC QLQ-H&N35, European organization for research and treatment of cancer head and neck quality of life questionnaire module; GC, gemcitabine, cisplatin; MDC, moderate dose/three weekly cisplatin; PF, cisplatin, fluorouracil; DP, docetaxel, cisplatin; 3D, three-dimensional radiotherapy; MEPFL, mitomycin, epirubicin, cisplatin, fluorouracil, leucovorin; CET, cisplatin, epirubicin, paclitaxel; SWOG, southwest oncology group; VAC, vincristine, Adriamycin, cyclophosphamide; VMAT, Volumetric Arc Therapy; FACT-HN, Functional Assessment of Cancer Therapy, Head & Neck cancer; MDASI-HN, MD Anderson symptom inventory, head and neck; FACT-NP, Functional Assessment of Cancer Therapy, Nasopharynx cancer; XeQoLS, University of Michigan Xerostomia-Related Quality of Life scale; (UW)-QoL, University of Washington Quality of Life Questionnaire*.

### Prospective Chemotherapy Studies

#### Chemoradiotherapy With or Without Neoadjuvant or Adjuvant Chemotherapy

The optimal integration of systemic treatment into the management of NPC has driven a large body of prospective clinical trials, both in endemic (2, 4, 6, 9–12, 22–24, 27–29, 31) and non-endemic regions (3, 25, 26, 30, 55, 56). NACT has emerged as the front runner, based on superior tolerability and compliance, but more importantly, has more consistently shown improvements in survival over concurrent cisplatin-based chemoradiotherapy (CRT) alone (9, 10, 22). Except where specifically mentioned, toxicity data has been reported from the perspective of the clinician.

The additional increase in toxicity from NACT appears to be manageable and limited to the treatment period, or shortly thereafter, with no differences in clinician-reported late toxicity. As anticipated, NACT results in higher rates of acute hematological toxicity, which in most cases is transient (9–11, 23, 24). While not a consistent finding, some studies report higher rates of nausea and/or vomiting (9–11, 23, 24) and severe mucositis (24) during the RT phase of treatment following NACT than without it. The data from these studies currently suggest that severe late toxicity (≥grade 3) is not enhanced with the addition NACT, however longer term reports are needed to confirm this finding (Table 2) (9, 12, 22–24). One of the concerns about combining cisplatin induction chemotherapy with concurrent cisplatin and radiation is the potential for increased toxicity related to the cumulative dose of cisplatin e.g., peripheral neuropathy. Indeed a higher rate of grade 1–4 peripheral neuropathy was reported in the gemcitabine-cisplatin induction study (9). In this trial there was also a higher incidence of acute nephrotoxic events. No difference in ototoxicity was reported, though it is possible that clinician reported late cisplatin side effects may underestimate the incidence.

**Table 2:** Selected studies reporting clinician-rated late toxicities (grade ≥3).

**Study**	**No**.	**Treatment**	**RT**	**FU**	**Any ≥G3**	**Xerostomia**	**Dysphagia**	**Hearing**	**TLN**	**CN**	**Notes**
**PROSPECTIVE CONCURRENT CRT** **±** **NEO/ADJUVANT CHEMOTHERAPY**											
Li et al. (22)	480	HDC-CRT ± TPF induction	IMRT	5.9 y	9% (9 vs. 8%)	1% (0.8 vs. 2%)	NR	6% (6 vs. 6%)	0% (0 vs. 0%)	1% (2 vs. 1%)	No difference between arms
Tan et al. (23)	172	LDC-CRT ± GCP induction	IMRT 98% 2D 4%	3.4 y	NR	8% (11 vs. 5 NS)	NR	NR	NR	NR	No difference between arms
Zhang et al. (9)	480	HDC-CRT ± GC induction	IMRT	3.6 y	10% (11 vs. 9%)	3% (2 vs. 3%)	NR	6% (7 vs. 5%)	0.4% (1 vs. 0%)	0.8% (0.8 vs. 0.8%)	No difference between arms, except higher G1/2 peripheral neuropathy in induction arm
Hui et al. (12)	65	LDC-CRT ± DP induction	3D	3.0y FU	57% (65 vs. 50%)	NR	1.6% (4 vs. 0%)	10% (12 vs. 9%, NS)	NR	NR	No difference in late toxicity between arms
Hong et al. (24)	479	LDC-CRT ± MEPFL induction	IMRT 61% 3D 39%	6.0 y	15% (13 vs. 17%, NS)	2% (2 vs. 2%)	6% (4 vs. 7%)	NR	NR	NR	No difference between arms
Chen et al. (6, 27)	508	LDC-CRT alone vs. LDC-CRT + PF	IMRT 42% 3D 5% 2D 53%	5.7 y	24% (21 vs. 27%, p=0.14)	7% (6 vs. 7%)	NR	12% (11 vs. 13)	3% (3 vs. 3%)	2% (2 vs. 2%)	No difference in late toxicity between arms
**PROSPECTIVE RT ALONE** **±** **CONCURRENT WITH ADJUVANT STUDIES**											
Lee et al. (28)	172	RT alone vs. HDC-CRT + PF	2D 41% Mix 8% Conf 61%	10.7 (min 10y FU)	52 vs. 47%, *p*=0.20)	NR	1.7% (2 vs. 1%)	24% (27 vs. 20%)	1% (2 vs. 0.6%)	6% (7 vs.5%	Chemotherapy did not increase late toxicity
**OTHER PROSPECTIVE CHEMOTHERAPY STUDIES**											
Chen, Li et al. (5, 22)	230	RT alone vs LDC-CRT	2D	10.4 y	26% vs. 35% (NS)	0 vs. 0%	NR	15 vs. 13%	10 vs. 6%	11 vs. 12	No difference in late toxicity between 3D and IMRT
**PROSPECTIVE INTENSITY-MODULATED vs. 2D/3D RADIOTHERAPY STUDIES**											
Kam et al. (32)	60	IMRT vs. 2D	IMRT 50% 2D 50%	1 y assessment	NR	39 vs. 82%^*^	NR	NR	NR	NR	
Peng et al. (14)	616	IMRT vs. 2D	IMRT 50% 3D 50%	3.5 y	NR	0 vs. 2 (≥G3) 10 vs. 29 (≥G2)	NR	26 vs. 84% (*p* <0.001)	21 vs. 13% (*p*=0.01)	4 vs. 9 (*p*=0.02)	Comparison of toxicity (grades) not clear in late analysis; significant differences across multiple late toxicities and additional for trismus and neck fibrosis
**OTHER ENDEMIC STUDIES**											
Fang et al. (35)	68	100% CRT	VMAT 100%	4 y cumulative incidence	3%	0%	NR	3%	0%	0%	Single arm VMAT report
Tsai et al. (19)	242	CRT 66%	IMRT 41% Non-IMRT 59%	EORTC QLQ-C30	CTCAE v4.0						
Lee et al. (13)	1593	-	IMRT 28% 3D 45% 2D 27%	6.8 y (0.2–18.4 y)	NR	NR	NR	IMRT 17% 3D 19% 2D 10%	IMRT 1% 3D 2% 2D 3%	IMRT 2% 3D 2% 2D 5%	
**NON-ENDEMIC STUDIES**											
Tonoli et al. (39)	136	CRT 91%	IMRT 95% 3D 5%	At 3 y	NR	1%	6%	9%	NR	NR	
McDowell et al. (40)	107	93% CRT	IMRT	7.5 y	47%	1%	3%	43%	0%	10%	
Takiar et al. (41)	66	CRT 99%	IMRT 100%	3.2 y	49% (5 years)	2%	0%^#^	29%	14%	3%	
Yee et al. (45)	82	CRT 77%	3D 43% 2D 57%	9.2 y	6%	0%	0%	1%	1%	NR	
Lastrucci et al. (42)	25	CRT 64%	IMRT 16% 3D 21% 2D 63%	7.1 y	12%	0%	0%	0%	0%	0%	
Ghiggia et al. (44)	21	CRT 100%	NS	4.5 y	NS	19%	NS	NS	NS	NS	

*RT, radiotherapy; FU, follow up; G3, grade 3 toxicity; TLN, temporal lobe necrosis; CN, cranial neuropathy; high dose/three weekly cisplatin; CRT, concurrent chemoradiotherapy; TPF, paclitaxel, cisplatin, 5-fluorouracil; IMRT, intensity-modulated radiotherapy; NR, not recorded; LDC, low dose/weekly cisplatin, GCP, gemcitabine, carboplatin, paclitaxel; 2D, two-dimensional radiotherapy; NS, not stated; GC, gemcitabine, cisplatin; DP, docetaxel, cisplatin; 3D, three-dimensional radiotherapy; MEPFL, mitomycin, epirubicin, cisplatin, fluorouracil, leucovorin; PF, cisplatin, 5-fluorouracil; Volumetric Arc Therapy*.

* *grade 2-4 xerostomia only reported*;

# *2/66 (5%) required esophageal dilatation for esophageal strictures, but a feeding tube was not required*.

Only two of these prospective NACT studies have reported QoL (Table 3) (12, 23). The larger, from Singapore, randomized 172 patients between concurrent weekly cisplatin (40 mg/m^2^) with or without induction therapy using gemcitabine, carboplatin and paclitaxel (23). Almost all patients were treated with intensity-modulated radiotherapy (IMRT; 98%). The QLQ-C30 and QLQ-H&N35 modules were collected at baseline, day 8 of cycle 2 in the NACT arm, during CRT (week 4), at the end of CRT, and at 3, 12, 24, and 60 months after treatment. Global QoL was similar between the two arms at all corresponding time points. Transient (and clinically small) differences favoring the CRT alone arm were observed in the EORTC QLQ-C30 dyspnoea (24.3 vs. 15.3; *p* = 0.014) and diarrhea (15.2 vs. 9.3; *p* = 0.018) scales. Somewhat counterintuitively patients in the CRT alone arm reported higher scores for pain, swallowing problems and pain killer use during treatment, and reported worse social contact at 3 months. It is unclear if these differences were purely statistical as the size of the difference was not included in the report. In all cases, these differences were isolated to the acute treatment and recovery period and differences resolved with follow up. In the smaller study of 65 patients reported by Hui et al., patients were randomized to CRT (with 3D-RT) with or without cisplatin-docetaxel induction (12). In both arms, declines were seen in the QLQ-C30 and QLQ-H&N35 scores compared to baseline in the acute treatment period, which gradually returned to baseline over time, and differences in global QoL scores did not differ between the arms at the measured times points. A deterioration in physical functioning scores was more marked in those receiving NACT during the treatment period (mean change in scores −42.9 vs. −27.7, *p* = 0.0499), although this resolved with further follow up. In the symptom scales, appetite (mean change 18.6 vs. −5.3; *p* = 0.023) and constipation (mean change 24.5 vs. −3.8; *p* = 0.0075) scores were worse in the NACT arm compared to the CRT alone arm at 4 weeks after CRT, but there were no differences at subsequent follow up. Differences by arm in the QLQ-H&N35 were seen only in the nutritional supplement score at the 24 month assessment only (mean change score, 10.0 vs. −23.5; *p* = 0.025), favoring the concurrent only arm.

**Table 3:** Selected NPC studies reporting Quality of Life outcomes.

**Study**	**No**.	**Treatment**	**RT**	**FU**	**QoL**	**QoL findings**
**PROSPECTIVE CONCURRENT CRT** **±** **NEO/ADJUVANT CHEMOTHERAPY**						
Tan et al. (23)	172	LDC-CRT ± GCP induction	IMRT 98% 2D 4%	3.4 y	EORTC QLQ-C30 and H&N35	No difference in global QoL, GCP arm worse dyspnea and diarrhea during CRT; H&N35, worse pain swallowing use of pain killers in GCP during CRT and for social contact at 3 m
Hui et al. (12)	65	LDC-CRT ± DP induction	3D	-	EORTC QLQ-C30 and H&N35	No difference in global QoL; Physical functioning more deterioration, appetite and constipation worse at 4 months in induction arm; all resolved with longer follow up; Only difference in H&N35 was nutritional supplement use in induction arm at 24m post treatment
**OTHER PROSPECTIVE CHEMOTHERAPY STUDIES**						
Lee et al. (2)	109	LDC vs. HDC CRT (+ adjuvant)	IMRT 74% 2D 16%	Last QoL at 12 m	EORTC QLQ-C30 and H&N35	Patients on weekly regimen showed better PF, EF and SF than the triweekly group and reported less appetite loss 3 weeks post treatment; Worse speech, social contact and sticky saliva in triweekly group; No differences at 3 and 12 m post treatment except dry mouth (3 m) and SF (12 m) post CRT
Pow et al. (33)	46	IMRT vs. 2D	IMRT 52% 2D 48%	Longitudinal assessment baseline, 2, 6 12 m post RT	EORTC QLQ-C30 and H&N35	No difference between IMRT and CRT global QoL at any point; at 12 m both global QoL scores = 64; No differences between baseline and 12 m post treatment, except global QoL, RF, EF were higher and insomnia lower than baseline in both groups; most of the domains showed improvement from 2 to 12 months. Role-physical, bodily pain, and physical function domains better in IMRT cohort
**OTHER ENDEMIC STUDIES**						
Fang et al. (17)	237	28% CRT	Conventional 64% (2D 26%, 2D + 3D boost 38%) Conformal 36% (3D 14%, IMRT 22%	-	EORTC QLQ-C30 and H&N35	Global QoL higher in conformal group (63 v51, *p* < 0.01) Conformal outcomes better across multiple points including PF, EF CF, SF functioning domains; 6/12 QLQ-C30 symptom scales and 12/13 H&N35 symptom scales On UVA, QoL better in higher educated, higher income, employed, conformal technique and less medical comorbidities
Fang et al. (34)	203	CRT 55%	IMRT 46% 3D 54%	Longitduinal Baseline, during RT and 3, 12, and 24 m post RT	EORTC QLQ-C30 and H&N35	12 and 24 months global QoL 61 and 62; IMRT better global QoL, fatigue, taste, dry mouth and feeling ill at 3 m, improved with time; No other medical, sociodemographic factors predicted worse QoL; General trend of deterioration followed by recover in most QoL scales from baseline to during, and then after RT
Fang et al. (35)	68	CRT 100%	VMAT 100%	Longitudinal: baseline, during RT, 3 m, 12 m	EORTC QLQ-C30 and H&N35	12 months global QoL mean score 78; Generally maximal decline in most scales from baseline to mid treatment with improvement thereafter; Chemotherapy or medical or sociodemographics factors did not predict QoL score
Hong et al. (36)	216	CRT 98%	IMRT 75% Conformal 25%	4.4 y (mean)	EORTC QLQ-C30	Mean global QoL 74; no difference by time since treatment; factors associated with better QoL were older age, higher education, higher anxiety and depression scores, worse dry mouth and fatigue, and higher disease stage
Fang et al. (18)	356	CRT 35%	IMRT 24% 3D 16% 2D+3D boost 30% 2D 30%	NR	EORTC QLQ-C30 and H&N35	Global QoL mean score 53; Age, gender, education, family income, CCI and RT technique associated with QoL; on multi-factor analysis education, family income and RT technique significant
Pan et al. (37)	106	CRT 48%	IMRT 56% 2D 44%	≥3 y (3.2–7.4 y)	EORTC QLQ-C30 and H&N35	Compared to CRT, RT alone patients reported better global QoL [77 vs. 68, *p* < 0.001)], PF, RF, EF; RT alone patients reported lower scores on selected symptom scales (fatigue, insomnia, financial problems, and weight gain); differences still apparent whether analyzed by RT technique
Tsai et al. (19)	242	CRT 66%	IMRT 41% Non-IMRT 59%	≥5 y	EORTC QLQ-C30	Mean global QoL 57; severe neuropathy, hearing loss and xerostomia associated with worse global QoL, all functional scales (PF, RF, EF, CF, SF, and some symptom scales.
Wu et al. (38)	192	CRT 23%	2D 34% Co-60 66%	3.6 y	Chinese SF-36	Most functional domains worse than general population. On multiple regression analysis medical comorbidities, monthly income, age, and T stage independently impacted global QoL
**NON-ENDEMIC STUDIES**						
McDowell et al. (40)	107	3% CRT	IMRT	7.5 y	FACT-HN	Mean total score FACT-HN 105.0 On UVA QoL associated with: marital status, employment status, time since treatment, chemotherapy, emotional distress, multiple clinician and patient-reported toxicities
Lastrucci et al. (42)	25	CRT 64%	IMRT 16% 3D 21% 2D 63%	7.1 y	FACT-NP XeQoLS	FACT-NP median 127; XeQoLS 31 younger patients better QoL; only xerostomia showed borderline association with global FACT-NP score (*p*=0.06)
Talmi et al. (43)	28	CRT 64%	2D	5.4 y (mean)	(UW)-QoL	Mean QoL score 4.2;
Ghiggia et al. (44)	21	CRT 100%	NS	4.5 y (mean)	EORTC QLQ-C30 and H&N35	Mean QoL score 74;

*RT, radiotherapy; FU, follow up; QoL, Quality of Life; LDC, low dose/weekly cisplatin; CRT, concurrent chemoradiotherapy; IMRT, intensity-modulated radiotherapy; 2D, two-dimensional radiotherapy; GCP, gemcitabine, carboplatin, paclitaxel; EORTC QLQ-C30, European organization for research and treatment of cancer core quality of life questionnaire; EORTC QLQ-H&N35, European organization for research and treatment of cancer head and neck quality of life questionnaire module; GC, gemcitabine, cisplatin; DP, docetaxel, cisplatin; HDC, high dose/three weekly cisplatin; 3D, three-dimensional radiotherapy; PF, physical functioning; EF, emotional functioning; SF, social functioning; RF, role functioning; CF, cogntivie functioning; FACT-HN, Functional Assessment of Cancer Therapy, Head & Neck cancer; FACT-NP, Functional Assessment of Cancer Therapy, Nasopharynx cancer; XeQoLS, University of Michigan Xerostomia-Related Quality of Life scale; (UW)-QoL, University of Washington Quality of Life Questionnaire*.

*y, year*.

In the single study to prospectively evaluate the addition of adjuvant chemotherapy (PF) following definitive chemoradiotherapy, Chen et al. found that severe late toxicity was similar between arms, with the exception of peripheral neuropathy, which was worse in the adjuvant arm, but the overall rate of severe toxicity (≥grade 3/4) was low (2 vs. 0.4%, *p* = 0.05) (6, 27).

Taken together, the published literature to date suggests that NACT or adjuvant chemotherapy is well-tolerated with manageable and time-limited toxicity compared to standard cisplatin-based CRT.

#### Prospective Studies of Chemotherapy Compared to Radiotherapy Alone

Radiotherapy as a single modality treatment is generally reserved for stage I disease (T1N0). While adding concurrent chemotherapy significantly improved survival in stage II patients (Chinese 1992 staging system) treated with 2D RT (5, 22), its necessity is under question in the IMRT era (57–59). In the prospective study by Chen et al., all patients were treated with 2D and the experimental arm consisted of weekly (30 mg/m2) cisplatin (5, 22). Patients in the CRT arm had a higher incidence of any severe acute toxicity (≥grade 3, 64 vs. 40%, *p* < 0.001), and a significant increase was observed for hematological toxicity, nausea and vomiting (9 vs. 0%, *p* = 0.001), and mucositis (46 vs. 33%, *p* = 0.04). At the 10 years follow up, there was no significant differences in late toxicity (RT 26 vs. CRT 35%). In a cross-sectional study which analyzed QoL outcomes in in stage II patients who had been treated with either RT alone or CRT, and a follow up time of at least 3 years, the authors reported that the addition of chemotherapy resulted in a detrimental impact on global QoL and a number of the functional and symptom scales from the QLQ-C30 and QLQ-H&N35 (37). When the analysis was restricted to patients treated only with IMRT, the authors reported a similar finding. In this study, the reported differences across the QLQ-C30 scores for the full and IMRT cohorts, which were in favor of the RT alone group would be considered small, with the exception of the financial problems item which would be considered of medium difference. In the IMRT alone cohort, there were some statistical differences favoring the RT alone group in the QLQ-H&N35 items, but only the less sexuality, nutritional supplements and weight gain items were in excess of ten points difference.

In the landmark studies which randomized patients to radiotherapy alone with or without concomitant and adjuvant chemotherapy, there was a predictable transient increase in severe hematological toxicity (3–5, 28, 29), mucositis (4, 5, 28), and nausea/vomiting (3–5, 28). Of these studies, the Hong Kong Nasopharyngeal Cancer Study Group NPC-9901 study published 10-year toxicity outcomes (28, 29). They reported no differences with respect to grade 3 or higher toxicities between the CRT and RT arms (52 vs. 47%, *p* = 0.20); and the majority of severe toxicity in both arms was attributable to ototoxicity, which was surprisingly similar between the arms (CRT 37 vs. RT alone 27%, *p* = 0.19). One interesting finding from this study was that severe late toxicity was higher in the chemotherapy arm at 3 years, a difference which dissipated with longer follow up. Numerically there was a shorter average latency time to developing a severe toxicity in the chemotherapy group which did not reach statistical significance (4.2 years vs. 4.7 years, *p* = 0.40). However, this latency period is quite informative when interpreting late toxicity data from other clinical trials, where follow up may not extend beyond 5 years.

#### Prospective Concurrent Studies—Three Weekly or Weekly Chemotherapy?

The optimal schedule of concurrent cisplatin in the definitive setting remains an unanswered question across the breadth of HNC subsites, including NPC. This question has only been addressed prospectively in NPC in a phase II study from the Korean Cancer Study Group (2). The study found no differences between clinician reported toxicity in the acute phase of treatment. While global QoL was not significantly different at any point during the study, patients treated on the weekly arm showed improved physical (*p* = 0.039), emotional (*p* = 0.019), and social functioning (p = 0.008) compared to the triweekly group at 3 weeks after completion of CRT, but these differences diminished at subsequent follow up. On the symptom scales (QLQ-C30 and H&N35), the triweekly group reported more appetite loss (*p* = 0.006) and problems with speech (*p* = 0.003), social contact (*p*-0.043) and sticky saliva (*p* = 0.019) than the weekly group. These differences again largely resolved (dry mouth had a persistent difference at 3 months) with longer follow up. Although both chemotherapy and RT for HNC can cause significant hearing loss and dysfunction, this is not captured in either of the EORTC modules. While non-NPC HNC series have reported worse ototoxicity with high dose (three weekly) compared to low dose (weekly) (60, 61), in this study it was not captured in the PROMs and only acute treatment-related toxicity was recorded; with only two patients reported as developing hearing loss, one from each arm.

#### Meta-Analysis of Chemotherapy in NPC Trials—MAC-NPC

The Meta-Analysis of Chemotherapy in Nasopharynx Carcinoma (MAC-NPC) collaborative group included an analysis of acute and late toxicity differences with the addition of chemotherapy to RT alone (7). This analysis predates the most recent practice-changing NACT studies. Mirroring results from the included studies, the addition of chemotherapy resulted in a significant increase in acute hematological toxicity and nausea/vomiting and mucositis. In addition, acute ototoxicity and more profound weight loss were also significantly higher with chemotherapy. While most of the individual studies did not identify an increase in late toxicities, the pooled analysis reported significantly higher rates of long-term hearing deficits (*p* = 0·00068) and a borderline increased risk of a cranial neuropathy (*p* = 0.052).

### Prospective Radiotherapy Specific Studies

#### Impact of Radiotherapy Technique Changes

The introduction of IMRT revolutionized NPC treatment, allowing both improved target volume coverage and reduced dose to adjacent organs at risk (OARs). At least three randomized controlled trials have addressed the benefit of IMRT compared to historical RT techniques, including 2D and 3D RT (Tables 1–3) (14, 32, 33). Peng et al. reported on the largest RCT, with 616 patients randomized to either IMRT or 2D RT (14). Local control favored the IMRT arm, with a corresponding reduction in the incidence of both acute toxicities, including xerostomia (≥Grade 2; 57 vs. 28%, *p* < 0.001) and hearing loss (89 vs. 47%, *p* < 0.001) and late toxicities (median follow up 3.5 years), including temporal lobe necrosis (TLN; 21 vs. 13%, *p* = 0.01), cranial neuropathy (9 vs. 4%, *p* = 0.02), trismus (14 vs. 3% *p* < 0.001), neck fibrosis (11 vs. 2%, *p* < 0.001), xerostomia (≥G1 99 vs. 40%, *p* < 0.001), and hearing loss (85 vs. 26%, *p* < 0.001). There was no QoL assessment in this study.

Two smaller prospective randomized studies specifically focused on the benefit of IMRT over 2D treatment with respect to salivary gland function (32, 33). Kam et al. reported lower rates of severe (clinician-reported) xerostomia in the IMRT arm (39 vs. 82%, *p* < 0.001), with corresponding higher rates of measured salivary flow (32). A xerostomia PROM was included, with a trend to improvement in the IMRT group. Pow et al. also reported that functional salivary flow showed significantly better recovery in the IMRT cohort (33). It is important to note that the mean doses delivered to the parotid in this study was higher than is generally used (where possible) in contemporary practice, with a mean ipsilateral and contralateral parotid dose of 42 (range 31–51Gy) and 41Gy (range 33–42 Gy), respectively. Using the EORTC QoL modules and the SF-36 questionnaire, IMRT-treated patients reported improved role-physical, bodily pain, and physical function scores at 1 year (all *p* < 0.05). Both groups reported dry mouth and sticky saliva, but better recovery of these xerostomia-type symptoms were observed in the IMRT cohort.

In a series form Taiwan, Fang et al. reported a longitudinal, but non-randomized comparison of 203 NPC patients, with any stage (I-IV), treated with either IMRT (*N* = 110) or 3D-RT (*N* = 93) (34). In this study, allocation was on the basis of clinician preference or linac availability. The EORTC QLQ-C30 and QLQ-H&N35 modules were assessed at baseline, mid treatment (week 4) and 3, 12, and 24 months after completion of treatment. The majority of patients completed all assessments (71.4%). As expected, maximal deterioration was seen during treatment which improved with subsequent follow up, and the benefit of IMRT over 3D-RT appeared to be limited to the early recovery period. The IMRT arm showed clinically meaningful better outcomes at 3 months for global QoL (56 vs. 44), fatigue (29 vs. 39), taste (22 vs. 35), dry mouth (49 vs. 59) and feeling ill (25 vs. 36); these differences diminished with ongoing follow up. From the same institution, a longer follow up of more than 5 years (median follow up: non-IMRT group 8.5 years, IMRT group 6.4 years) reported better long term toxicity experience for the IMRT cohort relative to the 3D-RT group across multiple domains of the QLQ-C30 and QLQ-H&N35, including moderate differences in global QoL, cognitive and social functioning, and fatigue on the QLQ-C30; and large differences in speech and dry mouth and moderate differences in most of the QLQ-H&N35 scales (16).

The experience and use of volumetric arc therapy (VMAT), is also increasing, and although there have been no direct clinical comparisons between standard IMRT and VMAT, planning studies have shown enhanced sparing of the parotids, brain stem and spinal cord is possible (62).

#### Adaptive Radiotherapy

Adaptive strategies in HNC offer the promise of reducing the degree of normal tissue irradiation based on early tumor response. In a replanning series by Yang et al., the outcomes of 129 IMRT-treated patients were compared. All patients were advised to have a repeat simulation scan either before the 15th fraction (12%), the 25th fraction (61%) or both (27%). However, only 86 were replanned (67%), while 43 (33%) refused, and consequently received the same plan throughout the treatment course (63). Two-year locoregional control favored the replanned arm (97.2 vs. 92.4%, *p* = 0.040), with similar survival (89.8 vs. 82.2%, *p* = 0.475). A significant benefit favoring the replanning group was reported on components of both the QLQ-C30 (global QoL, role and social functional scales and the dyspnea, appetite, constipation and diarrhea symptom scales) and the QLQ-H&N35 (speech, social contact, and teeth, mouth opening, dry mouth and sticky saliva) modules. Most of the differences were appreciable during the treatment and the early recovery period, with a diminishing difference with further follow up; for example, the differences in global QoL at 1 month post treatment (61 vs. 48), were of a medium sized clinical difference, while the difference at 12 months (82 vs. 78) is considered a trivial to small clinical difference (54). There was an imbalance in global QoL at baseline, favoring the non-replanning group (77 vs. 68), and while the non-replanned group returned to baseline with time, the replanned group actually demonstrated a positive shift from baseline, so the difference may be more significant than the 12-months results suggest. The diminishing difference with longer follow up was noticeable across most scales, with the exception of sticky saliva (24 vs. 17) and dry mouth (33 vs. 25), which both showed an ongoing higher symptom burden in the non-replanned group. While this is a potentially appealing strategy to reduce toxicity, adaptive treatment and replanning is resource-intensive. Further research regarding efficacy and efficient implementation is needed before adaptive treatment is likely to become routine.

#### Post-induction Response-Adapted Strategies

Adapting radiation dose and target volumes based on NACT response offers yet another strategy for reducing long-term toxicity and potentially improving QoL (64, 65). While better rates of local control have been achieved with a dose to the primary GTV ≥ 66.5 Gy (1), this may not be required for controlling microscopic disease in the setting of a complete response to NACT, when assessed by metabolic, radiological and clinical means. Yang et al. reported the results of a prospective randomized study including 212 patients with target volumes based on either pre (Arm A) or post induction (Arm B) imaging following a cisplatin doublet (paclitaxel or 5-FU) (64). Target definitions and doses used in this protocol are outlined in Table 4. Both the primary GTV (45 vs. 26 cm^3^, *p* < 0.001) and high dose CTV (367 vs. 305 cm^3^, *p* = 0.045) were smaller in Arm B. There were no differences in disease outcomes between these arms, either in the initial report (median follow up 35 months) (64), or in the subsequent 4 years update presented at ASTRO in 2019 (surprisingly, the presented data numerically favored the post-IC group) (66). There were no differences observed in acute clinician-rated toxicity, but lower rates of dry mouth were reported at 2 years in Arm B (grade 0-1/2-3: 68%/32% Arm A vs. 76%/24%; Arm B; *p* = 0.042). With respect to QoL, measured by the EORTC QLQ-C30, statistically significant differences in cognitive function (76 vs. 65) and lower pain scores (76 vs. 67) were reported, with the estimated clinical impact of these differences considered medium and small, respectively (54). In both arms the entire nasopharynx with a margin and the entire pre induction disease was treated to 64Gy/33#, and the usual intermediate risk area was included to 54Gy/33#, irrespective of response. Other series have also reported outcomes with full dose (70 Gy) to the post induction volumes and reduced doses (60 Gy) to the pre induction tumor volume (67, 68). Future strategies aimed at reducing toxicity will need to implement a strategy of reducing wide field treatment of the critical structures of the base of skull and brain to almost curative doses of radiation, capitalizing on how best to assess treatment response.

**Table 4:** Target volumes used in the study by Yang et al. (64).

**Target/Group**	**Group A (pre-induction)**	**Group B (post induction)**
Imaging timing	Pre-induction CT and MR	CT and MR at day 14, cycle 2 of induction (cisplatin + paclitaxel or 5-FU)
Pre-IC GTVnx	Clinical + imaging findings pre-IC;	Clinical + imaging findings pre-IC;
Post-IC GTVnx	N/A	Clinical + imaging findings post-IC;
GTVnx (includes primary and retropharyngeal nodes)	Pre-IC GTVnx	Post-IC GTVnx
P-GTVnx dose (GTVnx +3–5 mm)	70Gy/33#	70Gy/33#
CTV1 (subclinical disease)	Pre-IC GTVnx + 0.5–1 cm margin Whole nasopharyngeal wall 0.5cm margin under normal pharyngeal mucosa	Post-IC GTVnx + 0.5–1 cm margin Pre-IC GTVnx Whole nasopharyngeal wall 0.5 cm margin under normal pharyngeal mucosa
P-CTV1 dose (CTV1 + 3–5 mm)	64Gy/33#	64Gy/33#
GTVnd	Clinical + imaging findings of gross nodal disease post-IC Pre-IC ECE was assessed and included based in pre-IC imaging	Clinical + imaging findings of gross nodal disease post-IC Pre-IC ECE was assessed and included based in pre-IC imaging
P-GTVnd dose (GTVnd +3–5 mm)	70Gy/33#	70Gy/33#
CTV2	Pre-IC CTV1 Posterior nasal cavity Posterior maxillary sinus Pterygopalatine fossa Part of posterior ethmoid sinus Parapharyngeal space Skull base Clivus (whole/partial depending on involvement)	Post-IC CTV1 Posterior nasal cavity Posterior maxillary sinus Pterygopalatine fossa Part of posterior ethmoid sinus Parapharyngeal space Skull base Clivus (whole/partial depending on involvement)
Pre-IC CTV2	Elective nodal irradiation	Elective nodal irradiation
P-CTV2 (CTV2 + 3-5mm)	54Gy/33#	54Gy/33#

*CT, computed tomography; MR, magnetic resonance imaging; 5-FU, 5-Fluorouracil; IC- induction chemotherapy; GTVnx, nasopharynx gross tumor volume; P-GTV-nx, high dose primary planning target volume; CTV, clinical planning volume; P-CTV –planning target volume based on CTV; GTVnd, nodal gross tumor volume; P-GTVnd, high dose nodal planning target volume*.

Although large scale clinical trials are lacking, there is further suggestion in the literature that doses to gross disease below 70 Gy may provide adequate locoregional control in some patients. In a phase II study from the Peter MacCallum Center, 35 patients were treated with three cycles of induction epirubicin, cisplatin and 5-fluorouracil, followed by cisplatin-based chemoradiation (56). Radiation therapy was delivered with 2D planning to 60 Gy over 6 weeks at standard fractionation. After three cycles, 30/35 (86%) had a documented clinical and radiological (MRI/CT) response (2 complete, 28 partial). At a median follow up of 43 months only two patients had a locoregional relapse; one regional relapse in the elective nodal region (50 Gy) and one local (and concomitant distant) failure where the upper level of the field was limited to 54 Gy to remain under optic chiasm tolerance. In the previously mentioned retrospective study by Ng et al. a significant difference in local and regional control was reported when at least 66.5 Gy was delivered to the respective GTVs using IMRT (1). However, less than half (41.5%) received induction chemotherapy, and GTVs were contoured according to pre induction volumes. Other series have analyzed control rates in patients who failed to complete the prescribed course of treatment. Wang et al. performed a propensity match analysis comparing 234 patients completing the prescribed 70 Gy course of radiotherapy with 32 patients who did not complete treatment due to severe acute toxicities (69). In the entire cohort, 163/266 (61.5%) received NACT. The median dose in the non-completion group was 63.6 Gy (range 53–67.8 Gy) and the authors reported no difference in 5-years locoregional failure free survival (LRFS) between those receiving the reduced dose or the prescribed dose (92.5 vs. 91.7%; *p* = 0.863). Furthermore, radiation dose was not a significant variable in a univariate analysis of prognostic factors for LRFS. A smaller series (n=19) by Lu et al. concluded that patients who received a minimum of 54 Gy had a 5-years LRFS of 100% (70). However, this was a subset of only nine patients, and further details reported that there were no local or regional relapses in the 5-years follow up period when a minimum dose of 60 Gy was given. While interesting and potentially hypothesis generating, retrospective series reporting on patients failing to complete treatment are limited by bias, small numbers and heterogeneity in chemotherapy regimens.

Dose reductions, particularly to the base of skull region will likely offer a more beneficial strategy to reduce the burden of toxicity in NPC survivors than other proposed approaches, such as a reduction of elective nodal volumes. Under the auspices of a clinical trial, dose and volume de-escalation based on NACT response, incorporating clinical, radiological (both structural and metabolic) and biological response (including EBV-titres) may provide a real opportunity to further improve outcomes for these patients.

#### Reducing Elective Nodal Volumes

Two regions that may be spared from routine elective irradiation include the submandibular (Ib) station and the low neck (Level IV/Vb). Level Ib involvement at diagnosis or as a site of subsequent failure is an infrequent occurrence, although the risk may be higher in the following situations: [1] where a level II node measures >20 mm or demonstrates radiological extracapsular extension; [2] where there are bilateral cervical LNs (N2); or [3] if the primary tumor extends into the oropharynx (71). While this is appealing strategy to reduce toxicity, the clinical impact is less clear. The main rationale for omitting level Ib is to reduce xerostomia, however Zhang et al. failed to demonstrate a statistically significant reduction in the frequency of grade 2 or higher xerostomia in 904 low risk patients whether they had bilateral Ib sparing, unilateral Ib sparing, or inclusion of bilateral Ib (10 vs. 14 vs. 18%, *p* = 0.06). In optimizing dose reduction to the submandibular gland, the tradeoff may be an increase in dose to other xerostomia-critical organs such as the parotid or oral cavity (72). While a dose constraint of <39 Gy has been proposed for the submandibular gland, based on improvements in stimulated and unstimulated salivary gland flow in non-NPC cohorts, it can be challenging to meet this when there is gross disease at level 2, and mean doses ≥50 Gy are generally expected (73). Although there have been studies looking at omitting the low neck in selected case of NPC, there is little comparative data at present to suggest improved clinical outcomes with this approach (58, 74).

### Particle Therapy

Particle therapy offers the potential to reduce doses to OARs in HNC treatment (75, 76) and planning studies in NPC have shown improved metrics, with respect to both target coverage and OAR doses (77–80). However, clinical reports on efficacy and toxicity are limited (79, 81, 82). In a small retrospective series of 10 patients treated with intensity-modulated proton therapy (IMPT) from MD Anderson, Lewis et al. reported no late grade 3 toxicities (79); and grade 1 and 2 xerostomia were reported in 6 and 1 patient/s, respectively. The median follow up was only 24.5 months, shorter than the average latency time to development of severe toxicity (29). A case matched series from that institution (10 IMPT and 20 IMRT cases), reported lower rates of gastrostomy tube insertion during treatment in patients treated with IMPT, 65 vs. 20% (*p* = 0.020) (80). Results from a prospective phase II study from Massachusetts General Hospital are awaited, as to date this has only been presented in abstract form (81). In the abstract report, grade 3 or higher late hearing and weight loss was reported in 29 and 38% of patients, respectively, and 1/23 patients remained PEG dependent at 12 months.

#### Target Volume and Treatment Planning Heterogeneity

While the above studies have highlighted a number of different strategies focusing on reducing toxicity, two large sources of heterogeneity in the treatment planning process which may significantly impact toxicity are variations in target volume contouring and what constitutes an acceptable radiotherapy treatment plan (83–85). While the pioneering work of Peters et al. eloquently demonstrated the detrimental impact of poorly compliant radiotherapy treatment plans on long-term survival in HNC patients (86), a less obvious sin is that of over-contouring, which while resulting in locoregional control, may labor survivors with unnecessary morbidity. The findings from Peters et al. have spawned a generation of international consensus guidelines for HNC contouring and dose prioritization, including recommendations for NPC (87, 88), and while they may have reduced the variation to some degree, they have not completely mitigated it (85). In addition, an increasing recognition of the importance of HNC case volume and expertise (89, 90) and the value of peer-review in clinical practice (91, 92) will have hopefully further reduced this variation. However, with the recent number of immunotherapy trials opening, there has been a concerning trend in the number of trials failing to include a comprehensive radiotherapy quality assurance program (93). Clinician variability in both contouring and the variations accepted in radiotherapy treatment plans are arguably the most significant factors in determining long-term treatment toxicity, reducing the benefit of other toxicity-sparing measures mentioned above, and further ways to ensure the delivery of optimal treatment plans is required.

### Toxicity and Quality of Life Reports From Non-endemic Areas

The majority of studies reporting toxicity and QoL arise from NPC endemic regions, with a few studies reporting from non-endemic regions (Tables 1–3).

A prospective study of 136 patients treated at 12 Italian centers from 2008 to 2010 included patients mostly treated with IMRT (95%) and focused on clinician-reported toxicity (39). At 3 years follow up, hearing loss was the most common grade 3 toxicity (8.6%), followed by dysphagia (5.5%), periodontal disease (4.3%) and xerostomia (1.3%). The frequency of the most common grade 2 toxicities were xerostomia (23.4%), hearing loss (12.9%), and dysphagia (12.3%).

One of the more comprehensive studies from a non-endemic region comes from a cross-sectional study conducted at the Princess Margaret Hospital in Toronto (40). This study included clinician and patient-reported toxicity, a QoL measure, audiometry testing, basal endocrine screening (thyroid and pituitary) and a cognitive and neurobehavioral assessment. There were 107 patients enrolled at a median follow up time of 7.5 years (4.1–11.1 years) following IMRT. The highest rated patient-reported toxicities/problems from the MDASI-HN were dry mouth, mucous, swallowing/chewing, memory and teeth and gums. The prevalence of ≥grade 3 toxicities was 47% (50/107); mostly hearing loss (43%), with a much smaller prevalence (11%) of non-hearing grade 3 toxicities, including 11 patients (10%) with ≥grade 3 cranial neuropathies. One-quarter of patients reported moderate to high levels of fatigue. On univariate analysis, a number of factors correlated with worse global QoL, including social factors (living, marital and employment status), treatment factors (time since treatment, use of chemotherapy), emotional distress (depression or anxiety) and multiple clinician graded toxicities (hearing, ear discharge, dysphagia, trismus, aspiration, and cranial neuropathy). When correlating patient-reported toxicity, every item of the MDASI-HN PRO correlated with QoL, indicating that the presence of any significant symptom has capacity to adversely impact QoL.

In a series of IMRT-treated patients with T4 disease, the MD Anderson Cancer Center reported long term toxicity in 66 patients treated with a mean follow up of 66 months (1–124 months) (41). The actuarial rate of Grade 3 toxicity was 36% at 3 years and 49% at 5 years. Hearing problems were the most frequent severe toxicity, but the rate of severe toxicity not attributable to hearing at 3 and 5 years was 23 and 33%, respectively. Cranial or peripheral neurotoxicity was reported in 15/66 patients (23%) including two patients with grade 3 radiation-induced optic neuropathy; the remaining cases (13/15) had grade 2 toxicity. Radiological TLN was demonstrated in nine patients (14%), two with reported cognitive impairment; cognitive impairment was reported in an additional two patients without TLN.

### Factors Impacting on Long-Term Quality of Life

Factors which have been associated with QoL are summarized in Table 5. The studies show some conflicting findings, likely stemming from differences in patient populations, social constructs (particularly where sociodemographic factors are concerned) and variations in statistical methods to report predictive factors, including single or multi-factor models.

**Table 5:** Selected studies reporting factors associated with patient-reported quality of life.

**Factor/Study**	**Endemic**					**Non-endemic**
	**Fang et al**. **(****17****)**	**Fang et al**. **(****18****)**	**Hong et al**. **(****36****)**	**Wu et al**. **(****38****)**	**Tsai et al**. **(****19****)**	**McDowell et al**. **(****40****)**
QoL measure	EORTC QLQ-C30 global health score	EORTC QLQ-C30 global health score	EORTC QLQ-C30 global health score	General health measure as a surrogate for global QoL	EORTC QLQ-C30 global health score	FACT-HN total score
Stats analysis	Univariable analysis: categorical chi-squared; multivariable logistic regression	GLM-MANOVA one factor and multifactor models	Multiple linear regression	UVA (ANOVA) and MVA (multiple stepwise regression)	GLM-MANOVA one factor and multifactor models	univariable linear regression
**SOCIAL FACTORS**						
Sex	No	Yes (1F only)	No	Yes (UVA only)	Yes (1F only)	No
Age	No	Yes (older better; 1F only)	Yes (younger worse)	Yes (older better on MVA and UVA)	No	No
Marital status	No	No	NR	No	No	Yes (divorce/separated worse than married/common law)
Living status	NR	NR	NR	NR	NR	Yes (living with others better than isolated)
Education level	Yes (higher education >12y better on UVA, not MVA (*p*=0.08)	Yes (higher educated better, both 1F and MF)	Yes (higher educated better)	No	Yes (both 1F and MF)	No
Employment status	Yes (employed better on UVA, not MVA)	NR	NR	No	NR	Yes (homemaker/caregiver and disability leave worse than full time employment)
Income	Yes (higher family income better on UVA and MVA)	Yes (higher family income better, both 1F and MF)	NR	Yes (higher income better QoL on MVA)	NR	NR
**PATIENT FACTORS**						
Medical comorbidities	Yes (presence comorbidity worse on both UVA and MVA)	Yes (1F only)	NR	Yes (lower better QoL; UVA and MVA)	NR	NR
Depression	NR	NR	Yes (higher worse QoL)	NR	NR	Yes (higher worse QoL)
Anxiety	NR	NR	Yes (higher worse QoL)	NR	NR	Yes (higher worse QoL)
Recurrence Worry	NR	NR	No	NR	NR	NR
**TUMOR FACTORS**						
T-category	NR	NR	NR	Yes (lower T better QoL on UVA and MVA)	NR	NR
N-category	NR	NR	NR	No	NR	NR
Stage	No	No	Yes (higher stage worse QoL)	No	No	No (stage IVB worse than stage I)
**TREATMENT FACTORS**						
Time since treatment	NR	No	No	No	Yes (both 1F and MF)	Yes (better with longer FU)
Chemotherapy	No	No	NR	No	Borderline on 1F (*p* = 0.058)	Yes (none better than any)
Radiation tech	Yes (3D/IMRT better than 2D on both UVA and MVA)	Yes (both 1F and MF; IMRT better)	NR	No	Yes (both 1F and MF)	N/A (all IMRT)
**TOXICITY FACTORS**						
Clinician-reported	NR	NR	^*^ xerostomia and fatigue	NR	Neuropathy, hearing, xerostomia (both 1F and MF); Dysphagia and neck fibrosis (1F)	Hearing, ear discharge, dysphagia, trismus, dysarthria, aspiration, cranial neuropathy
Patient-reported toxicity	NR	NR	NR	NR		All items MDASI-HN correlated with QoL

*QoL, quality of life; EORTC QLQ-C30, European organization for research and treatment of cancer core quality of life questionnaire; FACT-HN, Functional Assessment of Cancer Therapy, Head & Neck cancer; GLM-MANOVA, general linear model multivariate analysis of variance; UVA, univariable analysis; ANOVA, analysis of variance; MVA, multivariable analysis; 1F, one factor analysis; NR, not recorded; MF, multifactor analysis; IMRT, intensity-modulated radiotherapy; MDASI-HN, MD Anderson symptom inventory, head and neck*;

* *toxicity was graded as yes/no*.

#### Sociodemographic Factors

##### Age

In some studies worse QoL has been observed in younger patients (42) while others have shown the contrary (18, 36, 38), and some have shown no association (40).

##### Sex

In some series, male survivors have been reported to have improved long-term QoL (18, 19), while others have reported that sex does not influence QoL (17, 36, 40).

##### Marital status/living status

Marital status has not be shown to influence global QoL in studies from endemic regions (17–19, 38). In contrast, the aforementioned series from Toronto reported that divorced or separated patients, but not single or widowed patients scored lower global QoL scores compared to cohabitating (married/common law/other) patients (40).

##### Education, and employment status/income

Education status (17–19, 36), employment status (17, 40, 94) and income have all been associated with improved global QoL in NPC cohorts (17, 18, 38).

#### Patient Factors

##### Medical factors

Where included, the burden of medical comorbidities has been linked with worse QoL in NPC survivors (17, 18, 38).

##### Emotional distress

Similar to series reporting for other HNCs, higher rates of anxiety and depression correlate with worse global QoL (95–98), in both endemic and non-endemic NPC series (36, 40, 44, 99–101). A complex interplay and association exist between QoL, toxicity and emotional distress, and a higher burden of toxicity has been shown to correlate with worse emotional distress and QoL in NPC survivors (40, 100).

#### Other

QoL is a complex construct which may be affected by many different factors that may or may not be captured in PROMs currently used in clinical research, including consultation satisfaction, optimism and worry (102).

#### Treatment Factors

##### Chemotherapy

The actual impact of chemotherapy on long-term QoL is difficult to quantify, given most prospective NPC studies have not included QoL assessments. The two NACT trials which included QoL assessments suggest that the addition of NACT may have a transient, but not long-lasting impact on QoL (12, 23). Based on the cross-sectional series included in Table 5, impact was either absent (17, 18, 38) or identified only in single factor models (19, 40) without accounting for confounding variables such as stage. In a study limited to patients with stage II NPC, it was reported that the addition of concomitant chemotherapy resulted in worse long term global QoL (54). When isolating these results to IMRT-treated patients only, the reported difference in EORTC QLQ-C30 global QoL score was 86 vs. 79, a difference which could be considered small or clinically insignificant (54).

##### Radiotherapy technique

Although not all studies have shown a significant improvement in QoL with IMRT (33), most studies have reported a positive impact (15–19). Adaptive strategies, including treatment replanning (63) and response-adaptive target volume delineation following induction chemotherapy are discussed above and have also been reported to improve QoL (64–66).

#### Toxicity Factors

Table 5 presents studies reporting the impact of individual toxicities on long-term QoL. Most have used clinician-rated toxicity and found a significant correlation between some, but not all the measured toxicities (19, 36, 40). PRO measures, however may be more reliable. In a study using the MDASI-HN tool, the mean symptom burden showed strong correlation with global QoL (FACT-HN total, *r* = 0.76, *p* < 0.001), and every item of the MDASI-HN inventory showed a very strong correlation with global QoL on univariate analysis, suggesting that the presence of any severe toxicity can adversely impact QoL (40).

### Treatment Toxicity—Where Can We Further Our Understanding?

Following many years of clinical trials data there is still much to understand. This article has highlighted past reliance on clinician-reported toxicities and the presence of “severe” or grade 3 or higher toxicity. This approach does not discriminate the varying effects of individual toxicities on the patient (not all grade 3 toxicities will impact patients to the same degree), and may fail to direct enough attention to the significant impact that grade 1–2 toxicities can have on the long-term well-being of the patient. In terms of relying on clinician reports, it is well-appreciated that clinicians may under report both symptoms (46, 47) and their severity (48, 49) compared to patients. In the series reported above by McDowell et al. xerostomia was ranked “severe” (grade 3 CTCAE) by only 1% of clinicians, yet 49% of patients rated their problems with dry mouth in the severe range (≥7 on the MDASI-HN item) (40). This large discrepancy is only one example of where potentially we can further our understanding of toxicity, and future research should focus on what matters to patients. In addition to the well-established toxicities, such as xerostomia, dysphagia and hearing loss, it is worth highlighting additional toxicities requiring further research, such as neurotoxicity, endocrine dysfunction and fatigue.

#### Neurotoxicity

##### Cranial neuropathies

Cranial neuropathies can be catastrophic for the NPC survivor, heralding a significant decline in QoL (19, 40). The average latency period is between four and seven years after treatment, but shorter or longer intervals are reported (103–106), with few effective options to reverse or stabilize symptoms (107). In the IMRT era, radiation induced cranial neuropathies have been reported in up to 14% of patients (Table 6). Although any of the cranial nerves may be affected, the hypoglossal nerve is most frequently involved, owing to its tortuous course near the high dose region of the nasopharynx and upper neck (40, 106). A hypoglossal nerve palsy can remain stable or demonstrate insidious progression, rendering a patient dependent on a feeding tube. In the largest series, reported by Chow et al., T-stage and diabetes mellitus as well as the dose to the hypoglossal nerve (D1cc: <74 Gy 2.4% vs. ≥74 Gy 20.8%, *p* < 0.001) were all predictors for a hypoglossal nerve palsy (106). This highlights the importance of optimizing treatment plans, ensuring dose homogeneity around the base of skull and reducing hotspots, despite these being deemed as acceptable by current ICRU guidelines. Chemotherapy may be a factor: in the MAC-NPC analysis, there was a borderline increased risk of cranial neuropathies with the addition of chemotherapy to RT alone (11·4% vs. 8·7%, HR = 1·35 (1·00–1·82), *p* = 0·052) (7). The potential impact of NACT is unknown.

**Table 6:** Selected studies reporting cranial or hypoglossal neuropathies in NPC patients treated with IMRT.

**Study**	**No**.	**Median follow up (range)**	**Incidence CN**	**Incidence hypoglossal neuropathy**	**Notes**
Lee et al. (13)	444	NR ^*^	1.6%	NR	IMRT lower rates than 2D (*p*=0.01)
Peng et al. (14)	306^**^	3.5y (0.1-6.9y)	3.9%	NR	IMRT lower rates than 2D (*p*=0.02)
Zhang et al. (9)	480	3.6y (2.9-5.4y)	3.8%	NR	G1/2 1.7% G3/4 2.1% combined arms of study; difference between NACT and CRT
Li et al. (22)	477	5.9y (0.1-7.5y)	3.7%	NR	G1/2–2.3% G3/4–1.4% Combined arms of study; difference between NACT and CRT
McDowell et al. (40)	107	7.5y (4.2-11.1)	14% (late)	13%	G1–6% G2–4% G3–4%
Chow et al. (106)	797	8.1y	NR	8.7%	74% unilateral; 26% bilateral;

*CN, cranial neuropathy; NR, not recorded; IMRT, intensity-modualted RT; G1/2, grade 1 or 2 toxicity; G3/4, grade 3 or 4 toxicity; NACT, neoadjuvant chemotherapy; CRT, concurrent chemoradiotherapy*;

* *not reported for IMRT cohort separately, for entire study range was 0.2-18.2 years including 2D, 3D and IMRT*.

** *306 in IMRT arm of this study*.

##### Cognitive and neurobehavioral toxicity

TLN is a well-recognized toxicity following NPC treatment (Table 2). IMRT-treated patients appear to have a significantly reduced risk of developing TLN (13). TLN has generally been reported as either symptomatic TLN, where the patient has a constellation of symptoms including reduced cognitive function with radiologic temporal lobe changes, or asymptomatic/radiologic TLN, where there is imaging evidence of necrosis in the temporal lobes, but the patient appears to be functioning normally from the perspective of the clinician. Prior to IMRT, TLN was shown to correlate with poorer cognitive function (99, 108), however this association has not been clearly demonstrated in IMRT-treated patients, although there are only few reports available (40, 109–112). In a cohort of 102 patients with a mean age and time since treatment of 56 and 7.5 years, respectively, McDowell et al. reported 32% of patents scoring in the neurocognitively impaired range (112). Asymptomatic TLN was reported in 22% of patients, which did not correlate with either objective (MoCA) or subjective (MDASI-HN memory problems item) cognitive assessment. In this series, frontal dysfunction was also high when self-rated by either the patient or their family member. Clinically significant rates of dysfunction in the domains of apathy, disinhibition and executive functioning were reported by 48, 35, and 39% of patients and 66, 53, and 56% of family members (who were reporting on the patients function), respectively. This study highlights the importance of measuring what matters; TLN has been used as a surrogate marker for radiation-induced damage to the brain and the designation of whether a patient is “symptomatic” has been based on a crude clinician assessment. However, directly measuring the functional impacts of our treatments, such as cognitive and frontal dysfunction provides more relevant and patient-focussed information, which can be used to counsel and consent patients. Cisplatin has also been implicated in cognitive dysfunction following cancer treatment (113), although the individual impacts of concurrent and neo/adjuvant chemotherapy and RT on cognition in NPC treatment have not been well-quantified in the literature.

#### Endocrine Dysfunction

##### Hypothyroidism

Compared to other HNC populations, NPC patients appear to be particularly vulnerable to developing primary hypothyroidism following neck irradiation (114). In IMRT series, up to 69% of survivors may develop hypothyroidism (40, 115–118). The median latency period has been reported in the range of 1.8–3.1 years (114–116), but the risk increases with further follow up and has been reported in excess of 10 years following treatment (114). Given this is a reversible toxicity which may significantly impact the QoL of survivors, all patients where the lower neck is irradiated should undergo indefinite biochemical screening following treatment. The National Comprehensive Cancer Network guidelines suggest testing thyroid-stimulating hormone every 6–12 months, but do not make a recommendation of duration (119).

##### Pituitary dysfunction

In a meta-analysis of pituitary dysfunction in adult patients treated with cranial irradiation, the point prevalence in the pooled NPC studies was 0.74 (120). There are some limitations to this data and further research is needed to assess the true impact in the IMRT era, where the risk may be lower. One study which included basal screening reported a low rate of 1%, with a median follow up of 7.5y (4.1–11.1 y) (40). However, in other populations exposed to cranial irradiation hypopituitarism may develop many years after RT (120). There is a lack of data about the true prevalence of pituitary dysfunction in NPC survivors treated with IMRT and the absolute benefit of regular screening in this group is unknown.

#### Fatigue

Fatigue frequently ranks highly among NPC patient-reported toxicities (17–19, 40). Fatigue has many potential causes in the NPC survivor, including reversible causes such as poor nutritional intake, endocrine dysfunction and emotional distress, and irreversible causes such as a high treatment-related symptom burden or the presence of distant metastases. Efforts to exclude reversible causes should be undertaken where appropriate, as fatigue has been linked to worse QoL in NPC series (36, 40). In the two NACT series which included the QLQ-C30, long-term fatigue was not worse in the NACT arms (12, 23). Other factors, such as the volume of posterior fossa irradiated may also contribute, as this has been linked to higher rates of long-term fatigue (clinician reported) assessments in both NPC (121) and non-NPC HNC studies (122).

### Unmet Needs

There has been little targeted research ascertaining unmet needs in NPC survivors, although studies in the general HNC population may be broadly translatable. Unmet needs cover a variety of domains that may not have been addressed at any point through the patients' cancer journey (123, 124), and include physical, psychological, informational, activities of daily living, social, spiritual/existential, nutritional, dental, communication, sexual and financial needs as well as access to care (125). A variety of validated tools exist to ascertain the unmet needs of cancer patients (125–127). In non-NPC HNC populations, patients frequently identify having an unmet need. For example, in a study of HNC survivors at the Princess Margaret Hospital, 61% (96/158) of survivors reported at least one unmet need, and patients with worse QoL reported an increased number of unmet needs (128).

Some of these domains are better understood than others. For instance, emotional distress has been reported in patients with NPC, and its presence often correlates with lower QoL (36, 40, 44). Fear of cancer recurrence is often cited an unmet need in HNC populations and in the study by Hong et al., frequent worry of recurrence was present in 18.5% of NPC patients (36). Workplace rehabilitation needs following treatment are also attracting increasing interest in the HNC literature. For instance in a Canadian study, which mostly included a migrant population from endemic areas, only 62% of patients who were employed prior to diagnosis and who were within working age at study enrolment (≤ 65) were working (median post-RT 7.5 years) (94). One-third of those who were working also reported working fewer hours than before their diagnosis (median 14 h, range 4–30). Patients who were working were younger, had a lower symptom burden, self-reported less changes in their frontal function and had private health benefits.

### Conclusion and Future Directions

The outlook for patients with NPC has substantially improved over the last 30 years, as a consequence of concerted global research efforts and technological advancements in RT planning and delivery. Despite these improvements, many NPC patients will still develop significant long-term toxicities, negatively impacting their QoL. Many opportunities offer early promise for reducing the burden of toxicity including adaptive RT, response-adapted treatment planning, and the potential offered by NACT, particle and proton therapy. Early research suggests these approaches may be beneficial in reducing toxicity and improving QoL. In designing future clinical trials, the focus needs to shift from clinician-reported to patient-reported outcomes, including both toxicity and QoL assessments, which have been lacking in most large-scale prospective studies to date. While there are a number of patient-reported tools appropriate for use in NPC patients, judicious selection of tools is required to ensure all significant treatment-related toxicity is being adequately captured. Currently, there is no “all encompassing” single tool for use during the treatment and survivorship phase of NPC treatment. As we continue to move into the era of immune and targeted therapies, additional toxicities and tools may need to be incorporated. In future studies, we would recommend including long-term follow up, in excess of 5 years is needed to fully quantify the development of late toxicities. Where reported, results from clinical trials should focus on reporting meaningful clinical differences in QoL measures rather than statistical differences which may be of little significance to patients. Targeted research to reduce the burden of toxicities such as cognitive and hearing impairment remain areas for future research, and clarifying the unmet needs of NPC survivors in endemic and non-endemic regions provides further opportunity to improve the survivorship experience.

## Author Contributions

LM designed the concept of the review and wrote the first draft of the manuscript. All others contributed to the manuscript revision, read, and approved the submitted version.

## Conflict of Interest

The authors declare that the research was conducted in the absence of any commercial or financial relationships that could be construed as a potential conflict of interest.
